# Identifying the Influencing Factors of Depressive Symptoms among Nurses in China by Machine Learning: A Multicentre Cross-Sectional Study

**DOI:** 10.1155/2023/5524561

**Published:** 2023-05-15

**Authors:** Shu Li, Kristin K. Sznajder, Lingfang Ning, Hong Gao, Xinyue Xie, Shuo Liu, Chunyu Shao, Xinru Li, Xiaoshi Yang

**Affiliations:** ^1^College of Health Management, China Medical University, Shenyang, Liaoning Province, China; ^2^Pennsylvania State University College of Medicine, 500 University Drive, Hershey, PA 17033, USA; ^3^First Affiliated Hospital of China Medical University, 155 Nanjing BeiJie, Shenyang, Liaoning Province, China

## Abstract

**Background:**

Nurses' high workload can result in depressive symptoms. However, the research has underexplored the internal and external variables, such as organisational support, career identity, and burnout, which may predict depressive symptoms among Chinese nurses via machine learning (ML).

**Aim:**

To predict nurses' depressive symptoms and identify the relevant factors by machine learning (ML) algorithms.

**Methods:**

A self-administered smartphone questionnaire was delivered to nurses to evaluate their depressive symptoms; 1,431 questionnaires and 28 internal and external features were collected. In the training set, the use of maximum relevance minimum redundancy ranked the features' importance. Five ML algorithms were used to establish models to identify nurses' depressive symptoms using different feature subsets, and the area under the curve (AUC) determined the optimal feature subset. Demographic characteristics were added to the optimal feature subset to establish the combined models. Each model's performance was evaluated using the test set.

**Results:**

The prevalence rate of depressive symptoms among Chinese nurses was 31.86%. The optimal feature subset comprised of sleep disturbance, chronic fatigue, physical fatigue, exhaustion, and perceived organisation support. The five models based on the optimal feature subset had good prediction performance on the test set (AUC: 0.871–0.895 and accuracy: 0.798–0.815). After adding the significant demographic characteristics, the performance of the five combined models slightly improved; the AUC and accuracy increased to 0.904 and 0.826 on the test set, respectively. The logistic regression analysis results showed the best and most stable performance while the univariate analysis results showed that external and internal personal features (AUC: 0.739–0.841) were more effective than demographic characteristics (AUC: 0.572–0.588) for predicting nurses' depressive symptoms.

**Conclusions:**

ML could effectively predict nurses' depressive symptoms. Interventions to manage physical fatigue, sleep disorders, burnout, and organisational support may prevent depressive symptoms.

## 1. Introduction

As a result of the COVID-19 pandemic phase, there is a growing concern about the health of healthcare professionals, such as nurses, who are immersed in stressors including overloaded clinical tasks and nursing assignment. Nurses play an essential role in the healthcare system but are often exposed to high workloads and stressful environments, such as those that place them in proximity to dying patients [1]. Nursing work traits have created a greater need for nursing professionals on the frontline, leading to high levels of overwork, detrimental psychological consequences, and the deterioration of mental health [2], including increased depressive symptoms.

Depressive symptoms not only affect nurses' health but also negatively affect their work performance and the quality of the care provided [3–5]. Depressive symptoms substantially reduce an individual's quality of life and life satisfaction. Nurses are highly prone to psychological health problems, such as depression [6]. According to a meta-analysis, approximately 30% of nurses working during the COVID-19 pandemic outbreak suffered from psychological symptoms, including anxiety, stress, and depression, with prevalence rates of 37%, 43%, and 35%, respectively [7]. In China, nurses had low mental health statuses during the COVID-19 pandemic phase, with prevalence rates for anxiety and depression estimated at 18.1% and 34.4%, respectively. Meanwhile, nurses who cared for COVID-19 patients had an extremely high prevalence rate of depression, at 47.1% [8].

Most of the current studies on nurses' depressive symptoms have mainly emphasised the association between depressive symptoms and negative physical and psychological outcomes, such as perceived stress [9], burnout [10], chronic fatigue [11], sleep quality [12], medication error [13], and decreased quality of care received by patients [13]. In China, there is a lack of an efficient supervision system and organisational institution to help nurses to cope with negative psychological health; instead, there is a larger focus on the improvement of nurses' professional skills and abilities. Recently, more attention has been paid to nurses' career identity and organisational support to help nurses combat occupational stress, ease their burden, and prevent them from experiencing depression [14, 15]. Moreover, positive psychology theory has gained traction, with an emphasis on positive personal resources, such as recovery experience and resilience [16, 17]. Based on the NAM model [18], the identifying factors that affect nurses' well-being can be categorised into external factors (i.e., sociocultural, regulatory, business, and payer environment, organisational factors, and learning/practice environment) and internal (individual) factors (i.e., healthcare role, personal factors, and skills and abilities). Therefore, this study explores the influence of the following related factors on nurses' depressive symptoms: (1) internal (individual) features, including demographic characteristics (i.e., age, sex, and economic status) and internal personal features (i.e., recovery experience, resilience, and chronic fatigue); and (2) external features (i.e., environmental and organisation resources and work-demand related factors), such as organisational support, career identity, and burnout.

Machine learning (ML) has a strong data training ability and obvious advantages in building prediction models, which may be helpful for identifying nurses' depressive symptoms. Most of the previous studies have employed traditional linear regression methods to analyse the relationship between independent variables and depressive symptoms [9–11]. ML can solve the problem of multiple related factors and multicollinearity between variables. Furthermore, ML can ascertain the contribution ranks of the predictors and the comparative effects of independent factors on the dependent variables [19]. Some studies have confirmed that ML models can effectively predict depression-related problems [20–22]. However, few studies have used ML methods to identify the influencing factors of depressive symptoms among nurses [23]; thus, it is necessary to conduct a more detailed analysis. To develop evidence-based interventions that can reduce the impact of depressive symptoms among nurses, it is necessary to determine the specific contributions of external variables, such as work-related factors, and internal personal factors, such as positive psychological resources, as well as demographic characteristics.

Consequently, the purpose of this study was to (1) detect nurses' depressive symptoms by applying ML and (2) identify the predictors of nurses' depressive symptoms and provide a research basis for reducing nurses' negative psychological outcomes, so as to improve their quality of life and well-being.

## 2. Materials and Methods

### 2.1. Design and Sample

This study employed a multicentre cross-sectional design in Liaoning Province, Northeast China, from January 2022 to April 2022 during the phase of normalization of COVID-19 prevention and control. This study used multistage proportional random sampling to collect information from nurses. It randomly selected two general hospitals from each city (Shenyang, Fushun, Fuxin, and Liaoyang) in Liaoning Province; around 30.0% of the nurses in clinical departments were selected from each hospital. Approximately 1,500 clinical nurses were finally selected from 8 hospitals. A self-administered anonymous smartphone questionnaire (via the Wenjuanxing platform) was delivered to the nurses to evaluate their depressive symptoms and related factors. After excluding illogical answers and invalid responses, the response rate was 95.4%.

### 2.2. Participants

This study included 1,500 clinical nurses aged 18 years and above who were working in the 8 selected general hospitals in Shenyang, Fushun, Fuxin, and Liaoyang and could complete the online questionnaire via the Wenjuanxing platform. Nurses who had been diagnosed with or treated for a severe mental illness (e.g., bipolar disorder, schizoaffective psychosis, and paranoid psychosis) were excluded from this study.

In this study, the sample size was calculated based on the following formula:

(1) $$\begin{array}{c} N=\frac{Z^{2}P\left(1-P\right)}{d^{2}}=2.582*0.3*\frac{0.3}{0},0352=1141,\frac{N}{0.8}=1369\text{.} \end{array}$$

The prevalence rate of depressive symptoms among the nurses after the epidemic is around 30%, and Π = 30% was used as the basis to estimate the sample size. “*d*” is the allowable error. To ensure accuracy, *d* = 3.5%, for 95% confidence interval, *α* = 0.01 and *Z* = 2.58. The estimated sample size was 1,369, taking into account a 20% loss of follow-up rate. In the final draft of this study, about 1,500 questionnaires were collected, excluding unqualified questionnaires, and 1,431 valid questionnaires remained, resulting in a valid response rate of 95.4% (1431/1500).

### 2.3. Data Preparation

**Figure 1:**
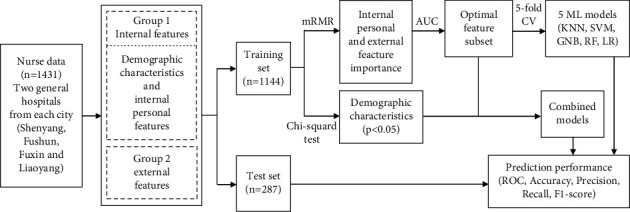
Flowchart of this study. CV: cross-validation and ML: machine learning.

This study excluded survey responses that were logically inconsistent. A total of 1,431 survey responses were obtained. The data were randomly divided into a training set (*n* = 1,144) and test set (*n* = 287) in an 8 : 2 proportion.  Figure 1 presents the detailed flowchart of this study.

### 2.4. Variables

This study used the Patient Health Questionnaire-9 (PHQ-9) [24] to measure nurses' depressive symptoms. The PHQ-9 is commonly used to measure depressive symptoms based on the Diagnostic and Statistical Manual of Mental Disorders. The scale is comparably sensitive and specific and includes nine items. A cut-off value score of ≥10 on the PHQ-9 indicates the existence of depressive symptoms. In this study, Cronbach's *α* coefficient of the PHQ-9 was 0.924. A detailed description of the questionnaire is provided in supplementary materials. This study categorised the predictors affecting the nurses' psychological health as internal and external features. Internal features included demographic characteristics (i.e., age, sex, marital status, income, and chronic disease) and internal personal features (i.e., coping styles, recovery experience, resilience, sleep quality, chronic fatigue, and perceived stress); external features included organisational support, career identity, and burnout.

Chronic fatigue had three dimensions: physical, affective, and cognitive [25]. Recovery experience had four dimensions: psychological detachment, relaxation, mastery, and control [26]. Coping had three dimensions: problem-focused coping, emotion-focused coping, and avoidant coping [27]. Burnout had three dimensions: exhaustion, cynicism, and professional efficacy [28]. Professional identity had seven dimensions: grasp, consistency, significance, self-efficacy, self-decision, organisational influence, and individual influence [29]. The remaining features contained only one dimension. In addition to these subfeatures, a total score was calculated for each class, yielding a total of 28 features.

This study simultaneously considered the sum score of these scales and their dimensions in analytic models to accurately explore the influencing factors of depressive symptoms among nurses.

### 2.5. Model Establishment and Performance Evaluation

This study implemented the following three steps to identify the important features of depressive symptoms among nurses. First, the variables describing demographic characteristics were analysed by using the chi-square test. It then used maximum relevance minimum redundancy (mRMR) to initially screen for the internal personal and external factors on the training set. mRMR combines two indicators to evaluate the importance of features: one maximises the correlation between features and variable classifications, the other minimises the redundancy between the features. In this study, the chi-square test calculated the correlation between the internal personal and external features and depressive symptoms. This study used Pearson's correlation coefficient to calculate the redundancy between the internal personal and external features. All internal personal and external features were sorted by importance.

Subsequently, this study conducted five ML algorithms using a five-fold cross-validation strategy to establish the models on the training set. This study used five ML algorithms: k-nearest neighbours (KNN), Gaussian Naive Bayes (GNB), support vector machine (SVM), random forest (RF), and logistic regression (LR). To discover the appropriate feature dimensions, this study gradually included the internal personal and external features according to the importance ranking to locate the optimal feature subset and identify whether there were depressive symptoms. By considering the five ML model results, this study obtained the smallest feature subset with the largest area under the curve (AUC) of the models. Then, this study evaluated the performance of the test set. Finally, the significant demographic features on the training set were added to the optimal internal personal and external features subset to establish combined models. Similarly, it evaluated the combined models' performance on the test set. Furthermore, this study used a univariate LR to further compare the predictive abilities of the important demographic characteristics and optimal internal personal and external features to determine the depressive symptoms and validate the abilities on the test set. This study compared the receiver operating characteristics (ROCS) of the demographic characteristics and internal personal and external features using the DeLong test.

To obtain the best model prediction performance, some interacting hyperparameters need to be tuned. The radial basis function was used as a kernel function of the SVM model, and a cross-validation grid search method was applied to find the best hyperparameters of the SVM model *γ* (from 1*e* − 03 to 1*e* + 03, number = 12) and C (from 1*e* − 04 to 1*e* + 04, number = 12). The random search method was used to find the best hyperparameter of the RF model and avoid overfitting, including the number of estimators (from 80 to 120, number = 5), the maximum depth of the tree (from 2 to 8, number = 3), minimum number of samples required to segment nodes (3, 5, or 10), and minimum number of samples per leaf node (5, 10, or 15). Stepwise LR was used to analyse depressive symptoms related features. The number of neighbours selected in the KNN model was 3, 5, 7, and 9. The GNB classifier used the default parameters to build the model. Hyperparameters adjustment results are provided in supplementary materials, Table S1.

### 2.6. Data Analysis

All statistical analyses were performed using Python (Version 3.7.3). The level of statistical significance was set at *P* < 0.05. In addition to the stepwise LR using the Statsmodels package, other ML methods used the scikit-learn package. The basic configuration of the computer for statistical analysis is as follows: CPU, Intel (R) Core (TM) i7-9700; RAM, 32 GB; and Operating system, Windows 10.

### 2.7. Ethical Statement

This study was implemented in accordance with the Helsinki Declaration (1989) and was approved by the Ethics Committee of China Medical University (ID: 2020048). The participants were voluntary and anonymous. They were well-informed of the aims and contents of this study and provided signed informed consent before the survey.

## 3. Results

### 3.1. Demographics Characteristics

Table 1 shows that out of the 1,431 nurses, 456 (31.9%) have depressive symptoms. The ages of the nurses range from 18 to 57 years. The majority are female (97.8%) and married (78.1%). Approximately, 64.5% receive an income of 3,000–6,000 yuan, and 23.5% suffer from a chronic disease.

In the training set, when compared with nurses who have no depressive symptoms, depressive symptoms demonstrate statistically significant differences in prevalence rates based on income, marital status, and chronic disease, which may indicate that unmarried nurses with low incomes and chronic diseases tend to suffer more often from depressive symptoms.

### 3.2. Construction of Optimal Feature Subset

Table 2 shows the importance of ranking of the internal personal and external features obtained from the mRMR. When the features dimension is five, the AUC of each model has the largest value. Ultimately, sleep quality, chronic fatigue, exhaustion, physical fatigue, and organisational support comprise the optimal feature subset that distinguishes depressive symptoms from no depressive symptoms.

Table 3 shows the evaluation indicators of the five models based on the optimal internal personal and external features. Each model has a good prediction performance based on the test set. This indicates that internal personal and external features can predict nurses' depressive symptoms.

### 3.3. Combined Models Establishment

**Figure 2:**
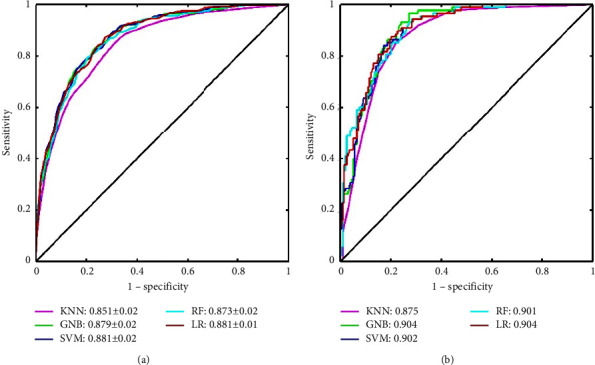
ROC curves of five combined models for nurses' depressive symptoms. (a) ROC curves for the model in the 5-fold cross-validation on the training set and (b) ROC curves for the model on the test set.

After adding income, chronic disease, and marital status to the optimal feature subset, the performance of the five combined models slightly improves. The AUC and accuracy increase to 0.904 and 0.826 on the test set, respectively. Compared with the other models, the LR model is more stable. Notably, only six features are included in the LR model; marital status and physical fatigue are excluded. These results suggest that sleep quality, chronic fatigue, exhaustion, organisational support, income, and chronic disease are the most important features for identifying nurses' depressive symptoms. For the LR model, the *P* values of the included variables are shown in supplementary material Table S2.  Table 4 shows the evaluation indicators of the five combined models on the training and test sets, and Figure 2 shows the ROCs of the combined models.

**Figure 3:**
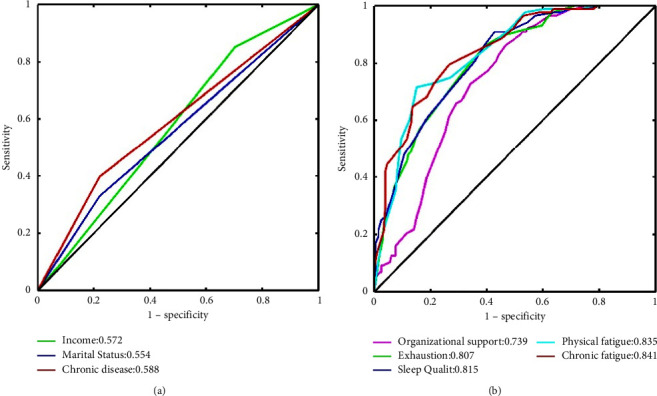
ROC curves of each feature for nurses' depressive symptoms on the test set. (a) ROC curves for the significant demographic features and (b) ROC curves for the optimal work-related and internal personal features.

Figure 3 shows the ROCs of the univariate analysis of the test set. The AUCs of the optimal internal personal and external features are much higher than those of the demographic characteristics, and chronic fatigue has the best prediction performance. According to the DeLong test results, there are no significant differences in ROC between organisational support and the demographic characteristics (*P*=0.068–0.237); however, the ROCs of the other features were significantly different (*P* < 0.001–*P*=0.016). These results suggest that internal personal and external features are more effective than demographic characteristics for predicting nurses' depressive symptoms.

## 4. Discussion

To the best of our knowledge, this study is the first to use ML to predict the risk factors of depressive symptoms among nurses in Northeast China. This study found that nurses had severe depressive symptoms, with a prevalence rate of 31.9%. This prevalence rate was much higher than that of a study conducted in Iran (17.8%) [30] and Li's study, which found that 26.2% of the nurses suffered from depression during COVID-19 isolation [31]. However, the current study's results were slightly lower than those of a study conducted in Sichuan Province and Wuhan City [8], which revealed that depressive symptoms had a 34.3% prevalence rate among nurses at the outbreak of COVID-19 and a 55.0% rate among frontline nurses at the beginning of the COVID-19 pandemic [32]. As a high-stress group, nurses are prone to negative emotions, such as depression, due to the specificities and limitations of their work environment, which, in turn, affect nurses' mental health and work quality. During the COVID-19 pandemic phase, nurses not only undertook heavy work tasks due to unprecedented workloads, but also faced the risk of infection, which could have triggered complex psychological stress responses and resulted in the development of depressive symptoms [32].

This study used five ML methods to identify the most important predictors of depressive symptoms among nurses. It achieved good predictive results: the LR model was the best predictor, with an AUC of 0.904 on the test set, while the KNN model was the worst predictor, but the AUC was also 0.871. The ML model results exceeded those of Zhou et al., who used four ML models to predict nurses' depressive symptoms during COVID-19 phase in China (AUCs: 0.785–0.829) [23]. Therefore, ML methods are feasible for predicting depressive symptoms among nurses, and the proposed predictive factors are reliable. Most of the previous research on predicting nurses' mental health using ML has focused on clinical characteristics or workplace factors and has highlighted health-related predictors and the importance of optimising workplaces [23, 33]. This study examined nurses' positive psychology resources, coping styles, organisational supportive resources, and career identity to predict depressive symptoms; these predictors can be conceptualised to improve nurses' psychological health. Overall, more attention should be paid to nurses' depressive symptoms, and effective measures should be taken to optimise their mental health status.

This study found that ML was an effective tool for predicting the most meaningful and distinctive features of depressive symptoms among Chinese nurses. A previous study by Havaei et al. used ML algorithms to predict the impact of work-related factors on nurses' mental health with significant beneficial results [33]. In the current study, the nurses' demographic characteristics, including marital status, income, and chronic disease were associated with their depressive symptoms, which concurs with the results of the previous studies [34]. More importantly, in the current study's ML models, the internal personal and external features could better explain most of the depressive symptoms among nurses than the demographic characteristics. Moreover, five key external and internal personal features could significantly predict depressive symptoms, including sleep disturbance, exhaustion due to burnout, chronic fatigue, physical fatigue, and perceived organisation support. By comparing the five depression prediction models, this study found that the LR model had the best performance for predicting depressive symptoms, and the strongest predictor was sleep quality. However, physical fatigue was excluded.

The results also showed that sleep quality, exhaustion due to burnout, chronic fatigue, and physical fatigue were positively associated with depressive symptoms. Perceived organisational support, as a protective factor, was associated with depressive symptoms, which was consistent with the previous studies [12, 35–37]. Compared with traditional LR, the multicollinearity problem between variables can be solved using ML algorithms, and more effective predictors can be screened out [38]. This study differs from the previous studies that have employed ML algorithms as it incorporates the total score of the scales into the prediction model simultaneously with each scale dimension to effectively understand the distinctive contributions of each dimension and the total effect of the overall factor [33, 39]. Thus, the fractional dimension and the total effect of the feature are not interchangeable, meaning that if you obtain a high-dimensional score of the feature, you can simultaneously obtain a high total score of the feature. Therefore, this study simultaneously considered both the fractional dimension and the total effect of the feature on depressive symptoms.

Sleep quality was a critical predictor of nurses' depressive symptoms; this is in agreement with the previous studies that have shown that sleep disorders impact an individual's ability to tackle work tasks, which, in turn, negatively impacts job performance, reduces work productivity, and ultimately affects quality of care, thereby increasing the risk of depression [12]. The research has also confirmed that there is a strong association between sleep quality and depression [40]. Sleep disturbances may cause changes in epigenetic characteristics, personality, and neurobiological functioning, which are typical risk factors of depression. Moreover, sleep disorders are accompanied with fatigue; therefore, subsequent mental impairments, such as depressive symptoms, may occur.

In this study, both the chronic and physical fatigue dimensions were crucial predictors of depressive symptoms. Fatigue is a common problem that affects healthcare professionals, especially during the COVID-19 pandemic phase in China. The consequences of chronic and physical fatigue can impair an individual's recovery functioning and vigour at different levels and can interrupt harmonious relations between family members and work [41]. Chronic fatigue caused by a depleted ability to restore and recover physical and mental health could increase an individual's susceptibility towards developing depressive symptoms [42]. Moreover, the longer fatigue lasts and the greater its intensity, the more it can impact performance in terms of daily activities and duties inherent to various professionals in social roles. Depressive symptoms could be further aggravated by chronic fatigue or physical exhaustion in stressful work surroundings. Some scholars have argued that fatigue and depressive symptoms have a high degree of overlap, while recent studies have suggested that chronic fatigue could be treated as an independent predictor of depressive symptoms. Therefore, fatigue should be managed to optimise nurses' mental health status [43].

This study found a strong association between exhaustion and depressive symptoms and revealed that the greater the level of burnout experienced, the more depressive symptoms reported by the nurses. There has been much attention on burnout among nurses, which has subsequently been identified as one of the occupational risks for nurses due to its high morbidity [44]. Many studies have found that all three dimensions of burnout are significantly associated with depression [45], while the current study found that only exhaustion due to burnout was a powerful predictor of depressive symptoms. Meanwhile, a study conducted in a Brazilian hospital noted that too many work tasks assigned to a nurse could lead to faster work rhythms and less time off work. Moreover, nurses must deal with many patients and their family members, which could enhance their vulnerability to stress-related disorders [46] and exacerbate depressive symptoms.

In addition, perceived organisational support was conversely correlated with depressive symptoms, as found in other studies [14, 15]. Nurses with a high level of perceived organisational support were less likely to experience depressive symptoms. This might be because nurses who perceive organisational support and feel that their work contributions are valued may be more likely to adopt an optimistic working attitude, which would positively impact their mental health [47, 48]. It has also been previously studied that perceived organisational support could relieve depressive symptoms in different populations, such as policemen [37, 49]. The less support perceived from organisations, the more severe depressive symptoms the nurses exerted. Supportive organisations can help nurses combat stressful work tasks, reducing the incidence of depressive symptoms. Organisational support has a beneficial effect on depressive symptoms, which helps nurses cope appropriately when confronting their working environment and thus can prevent depressive symptoms [50]. Nowadays, most hospitals underestimate the mental health demands put on nurses and the requisite supportive strategies to foster psychological health, resulting in widely varying levels of organisational support in various healthcare settings [51]. Organisational support interventions should help the nurses facilitate communication with the supervisors and leaders of the hospital and increase the nurses' participation in decision-making to improve the social support to combat depressive symptoms. At the organisational level, hospitals should decrease the working pace and workloads of nursing and increase the number of staff to ease work overloads and burnout. Medical institutions should develop strategies to incentivise enhanced organisational support, which will prevent depressive symptoms in the nursing population and ultimately improve the quality of care for patients.

The limitations of the study should be made an explanation. First, this study did not comprehensively consider other associated factors of depressive symptoms among nurses, such as fear of COVID-19, the time of COVID-19 phase, the work organisation, and work conditions including work climate. However, this study's results may have reference value for the future research on psychological interventions at the individual and organisational level. Second, this study's design was a cross-sectional survey so the causal relationship between variables could not be confirmed; therefore, further longitudinal studies should be conducted in this regard. Third, this study's results could be optimised through other various and efficient ML methods. Finally, the study was conducted in Northeast China, which may limit the generalisability of the results in different national and organisational contexts.

## 5. Conclusion

This study found that Chinese nurses in clinical settings suffered from severe depressive symptoms, and ML constituted a feasible approach to identify the predictors of depressive symptoms. Specifically, the LR model could successfully capture high-dimensional information on the risk factors of depression among nurses. Sleep disturbance, exhaustion due to burnout, chronic fatigue, and physical fatigue were important predictors of depressive symptoms. Furthermore, organisational support could relieve depressive symptoms. To prevent depressive symptoms among nurses, this study suggests to prioritise interventions to improve the management of fatigue, sleep quality, and exhaustion due to burnout, and emphasise organisational support to enhance nurses' work–life balance.

## 6. Implications for Nursing Management

By using ML to predict depressive symptoms in nurses, this multicentre cross-sectional study can help nursing managers identify psychological problems in nurses. ML prediction is a promising strategy for predicting depressive symptoms in Chinese nurses, and it provides evidence-based recommendations for preventing depressive symptoms. For internal personal features and external variables, the ML prediction model has high validity and effectiveness. This study supports the identification of nurses' mental health problems. Moreover, improving nurses' mental health contributes to the quality of care and patient satisfaction with healthcare services as well as playing an important role in strengthening nursing care and creating value for the healthcare system.

## Figures and Tables

**Table 1:** Analysis of no depressive symptoms and depressive symptoms on the training and test sets according to the patient demographic characteristics.

Demographic characteristics	Total (%)	Train set			Test set		
		No depressive symptoms (%)	Depressive symptoms (%)	*P* value^*∗*^	No depressive symptoms (%)	Depressive symptoms (%)	*P* value^*∗*^
*Age (years)*				0.2874			0.9027
≤36	867 (60.6)	460 (59.3)	231 (62.8)		123 (61.8)	53 (60.2)	
>36	564 (39.4)	316 (40.7)	137 (37.2)		76 (38.2)	35 (39.8)	

*Gender*				0.4287			0.3121
Man	31 (2.1)	20 (2.6)	6 (1.6)		5 (2.5)	0 (0.0)	
Woman	1400 (97.8)	756 (97.4)	362 (98.4)		194 (97.5)	88 (100.0)	

*Marital status*				**0.0113 ** ^*∗*^			0.0722
Married	1118 (78.1)	630 (81.2)	274 (74.5)		155 (77.9)	59 (67.1)	
Others	313 (21.9)	146 (18.8)	94 (25.5)		44 (22.1)	29 (32.9)	

*Income*				**<0.0001 ** ^*∗∗*^			**0.0272 ** ^*∗*^
<3000	113 (7.90)	55 (7.1)	37 (10.1)		14 (7.0)	7 (7.9)	
3000–6000	923 (64.50)	465 (59.9)	264 (71.7)		126 (63.3)	68 (77.3)	
>6000	395 (27.60)	256 (33.0)	67 (18.2)		59 (29.7)	13 (14.8)	

*Chronic disease*				**<0.0001 ** ^*∗∗*^			**0.0032 ** ^*∗∗*^
Yes	337 (23.5)	124 (16.0)	134 (36.4)		44 (22.1)	35 (39.8)	
No	1094 (76.5)	652 (84.0)	234 (63.6)		155 (77.9)	53 (60.2)	

Note:^*∗*^Chi-squared test for all demographics characteristics. ^*∗*^*P* < 0.05, ^*∗∗*^*P* < 0.01. Bold values indicate significant differences.

**Table 2:** The ranking of internal personal and external features importance obtained by the mRMR method.

Work-related and internal personal feature	Importance ranking
Organizational support	**5**
Sleep quality	**1**
Resilience	11
Physical fatigue	**4**
Affective fatigue	14
Cognitive fatigue	7
Chronic fatigue	**2**
Psychological detachment	27
Relaxation	16
Mastery	19
Control	8
Recovery experience	22
Problem-focused coping	24
Emotion-focused coping	28
Avoidant coping	17
Perceived stress	10
Exhaustion	**3**
Cynicism	6
Professional efficacy	21
Job burnout	12
Sense of grasp	20
Sense of consistency	9
Sense of significance	18
Sense of self-efficacy	23
Sense of self-decision	13
Sense of organisational influence	25
Sense of individual influence	26
Career identity	15

Bold values indicate important features of mRMR selection.

**Table 3:** The results of five models based on optimal internal personal and external features for depressive symptoms on the training and test sets.

Data sets	Model	Accuracy	Precision	Recall	F1-score	AUC
Training set	KNN	0.785 ± 0.03	0.779 ± 0.03	0.785 ± 0.03	0.777 ± 0.03	0.851 ± 0.03
	GNB	0.795 ± 0.02	0.802 ± 0.01	0.795 ± 0.02	0.797 ± 0.02	0.871 ± 0.02
	SVM	0.803 ± 0.02	0.799 ± 0.02	0.803 ± 0.02	0.792 ± 0.02	0.874 ± 0.02
	RF	0.787 ± 0.01	0.781 ± 0.01	0.787 ± 0.01	0.780 ± 0.01	0.870 ± 0.02
	LR	0.798 ± 0.01	0.794 ± 0.01	0.798 ± 0.01	0.790 ± 0.01	0.875 ± 0.02

Test set	KNN	0.798	0.791	0.798	0.788	0.871
	GNB	0.791	0.803	0.791	0.794	0.893
	SVM	0.808	0.803	0.808	0.799	0.894
	RF	0.815	0.810	0.815	0.810	0.892
	LR	0.801	0.795	0.801	0.803	0.895

**Table 4:** The results of five combined models for depressive symptoms on the training and test sets.

Data sets	Model	Accuracy	Precision	Recall	F1-score	AUC
Training set	KNN	0.796 ± 0.01	0.791 ± 0.01	0.796 ± 0.01	0.787 ± 0.01	0.851 ± 0.02
	GNB	0.807 ± 0.02	0.816 ± 0.02	0.807 ± 0.02	0.810 ± 0.02	0.879 ± 0.02
	SVM	0.798 ± 0.01	0.796 ± 0.01	0.798 ± 0.01	0.785 ± 0.01	0.881 ± 0.02
	RF	0.803 ± 0.01	0.798 ± 0.01	0.803 ± 0.01	0.795 ± 0.01	0.873 ± 0.02
	LR	0.811 ± 0.01	0.807 ± 0.01	0.811 ± 0.01	0.803 ± 0.01	0.881 ± 0.01

Test set	KNN	0.798	0.792	0.798	0.793	0.875
	GNB	0.824	0.831	0.824	0.825	0.904
	SVM	0.822	0.820	0.822	0.818	0.902
	RF	0.819	0.819	0.819	0.819	0.901
	LR	0.826	0.822	0.826	0.823	0.904

## Data Availability

The datasets used during the study are available upon reasonable request from the corresponding author.

## Additional Points

*Implications for Nursing Management*. ML is a promising strategy that can predict depressive symptoms and make evidence-based recommendations for interventions to prevent depressive symptoms among Chinese nurses.

## Supplementary Materials

Supplementary Materials S (detailed descriptions of the questionnaire), Table S1 (the results of tuned hyperparameters), and Table S2 (LR model for nurses' depressive symptoms).
