# Supplementary material for: Identifying the Influencing Factors of Depressive Symptoms among Nurses in China by Machine Learning: A Multicentre Cross-Sectional Study
Source: J Nurs Manag. 2023 May 15;2023:5524561. doi: 10.1155/2023/5524561 (PMC11918513; doi:10.1155/2023/5524561)
Supplement: Supplementary Materials — S (detailed descriptions of the questionnaire), Table S1 (the results of tuned hyperparameters), and Table S2 (LR model for nurses' depressive symptoms). [file 5524561.f1.docx]

**Supplementary material S**

**Measurement of Perceived Organizational Support**

Perceived organizational support was assessed by Perceived Organizational Support Scale (POS). The POS developed by Eisenberger (Eisenberger R et al., 2001), which had 9 items. The Cronbach α coefficient of the POS was 0.920.

**Measurement of Career Identity**

We employed the Nurse's Career Identity Scale to test nurses' **career identity** (Zhao H, 2010). The scale involves seven dimensions: sense of grasp, sense of consistency, sense of significance, sense of self efficacy, sense of self decision, sense of organizational influence and sense of individual influence.The Cronbach α coefficient of career identity in our study is 0.973.

**Measurement of Perception of Stress**

Perception of stress was measured by the Perceived Stress Scale (PSS), which was developed by Cohen(Cohen S, 1983). This scale consists of 10 items, each scored from 0 to 4, with higher levels of stress scoring. The Cronbach α coefficient of the PSS was 0.789.

**Measurement of Job Burnout**

Job burnout was measured with the Maslach Burnout Inventory-General Scale, which is the most widely used measurement tool of burnout (Maslach C, 1981). The Cronbach α coefficient of this scale was 0.914.

**Measurement of Coping Styles**

Coping styles was evaluated by means of an abbreviated version of the Cope Inventory (Brief COPE) (Carver CS, 1997). The Brief COPE consists of 28 items. This questionnaire used a 7-point hierarchical method to indicate the degree to which employees felt organizational support. Higher scores represent greater perceived organizational support. The Cronbach α coefficient of the Simplified Coping Style Questionnaire was 0.928, which indicates that the scale has good reliability.

**Measurement of Recovery Experience**

The Recovery Experience Questionnaire (REQ) was used to evaluate the levels of recovery experience(Sonnentag S,2007). This scale consisted of 16 items.The scale was divided into four dimensions: namely, psychological depression, relaxation, mastery, and control. The total score was summed across entry scores, with higher scores indicating higher levels of recovery experience. The Cronbach α coefficient of the REQ was 0.919.

**Measurement of Resilience**

Resilience was measured with the Ego Resilience 89 Scale (Block J, 1996), with scores ranging from 1 to 4 (1=“does not apply at all”; 2=“applies slightly, if at all”; 3=“applies somewhat”; 4=“applies quite strongly”).The total score is summed across entry scores, with higher levels of resilience being scored. The Cronbach α coefficient of the Ego Resilience 89 Scale was 0.933.

**Measurement of Sleep Quality**

Sleep Disturbance Short Form was used to assess sleep disturbance (Yu L, 2011). It has 8 items.The Cronbach α coefficient of the scale was 0.890.

**Measurement of Chronic Fatigue**

Chronic fatigue was evaluated byCancer Fatigue Scale (CFS), consisted of 15 items, which were scored on 5-point Likert scale (Okuyama T, 2000). The Cronbach α coefficient of CFS was 0.876.

**Reference**

Block, J., & Kremen, A. M. (1996). IQ and ego-resiliency: conceptual and empirical connections and separateness. *Journal of personality and social psychology*, 70(2), 349–361. https://doi.org/10.1037//0022-3514.70.2.349

Carver C. S. (1997). You want to measure coping but your protocol's too long: consider the brief COPE. *International journal of behavioral medicine*, 4(1), 92–100. https://doi.org/10.1207/s15327558ijbm0401_6

Cohen, S., Kamarck, T., & Mermelstein, R. (1983). A global measure of perceived stress. *Journal of health and social behavior, 24*(4), 385–396.

Eisenberger, R., Armeli, S., Rexwinkel, B., Lynch, P. D., & Rhoades, L. (2001). Reciprocation of perceived organizational support. *The Journal of applied psychology*, 86(1), 42–51. https://doi.org/10.1037/0021-9010.86.1.42

Maslach, C., & Jackson, S. E. (1981). The measurement of experienced burnout. *Journal of Occupational Behavior*, 2, 99 –113.

Okuyama, T., Akechi, T., Kugaya, A., Okamura, H., Shima, Y., Maruguchi, M., Hosaka, T., & Uchitomi, Y. (2000). Development and validation of the cancer fatigue scale: a brief, three-dimensional, self-rating scale for assessment of fatigue in cancer patients. *Journal of pain and symptom management*, 19(1), 5–14. https://doi.org/10.1016/s0885-3924(99)00138-4

Sonnentag, S., & Fritz, C. (2007). The Recovery Experience Questionnaire: development and validation of a measure for assessing recuperation and unwinding from work. *Journal of occupational health psychology*, 12(3), 204–221. https://doi.org/10.1037/1076-8998.12.3.204

Yu, L., Buysse, D. J., Germain, A., Moul, D. E., Stover, A., Dodds, N. E., Johnston, K. L., & Pilkonis, P. A. (2011). Development of short forms from the PROMIS™ sleep disturbance and Sleep-Related Impairment item banks. *Behavioral sleep medicine*, 10(1), 6–24. https://doi.org/10.1080/15402002.2012.636266

Zhao, H., Lu, T., Zhang, C., Onishi, M., Nagata, A., Kobayashi, H., & Kanda, K. (2010). Testing for reliability and validity of Chinese version of the nurse’s career identity scale. *Chin Nurs Manag. 10*(11):49-51.

**Table S1** The results of tuned hyperparameters

| **ML method** | **parameters of models** | **Work related and internal personal features models** | **Combined models** |
| --- | --- | --- | --- |
| KNN | n_neighbors | 10 | 10 |
| SVM | C | 6.579332 | 6.873817 |
|  | Gamma (γ) | 0.002848 | 0.001356 |
| RF | Number of estimators | 110 | 90 |
|  | Max depth of trees | 5 | 2 |
|  | Min samples split | 3 | 5 |
|  | Min samples leaf | 15 | 10 |
|  | Bootstrap method | True | True |

**Table S2** Stepwise logistic regression model based on combined features for nurses' depressive symptoms.

| **Variable** | **Coef .** | **P -value *** | **95%CI** |
| --- | --- | --- | --- |
| Constant | -1.3235 | <0.0001 | -1.5428 , -1.1494 |
| Sleep quality | 1.0783 | <0.0001 | 0.8072 , 1.2810 |
| Chronic fatigue | 0.8748 | <0.0001 | 0.5797 , 1.1431 |
| Physical fatigue | _ | _ | _ |
| Exhaustion | 0.3421 | 0.0014 | 0.1414 , 0.5683 |
| Organizational support | -0.4339 | <0.0001 | -0.5939 , -0.1824 |
| Marital Status | _ | _ | _ |
| Income | -0.2231 | 0.0110 | -0.3950, -0.0512 |
| Chronic disease | 0.2685 | 0.0006 | 0.1142, 0.4228 |
